# Fatigue Behavior of Dental Implants Affected by Peri-Implantitis-Related Bone Loss: Influence of Implantoplasty Evaluated Through In Vitro Testing and Finite Element Modeling

**DOI:** 10.3390/dj14060329

**Published:** 2026-06-01

**Authors:** Esteban Padullés-Roig, Darcio Fonseca, Juan Antonio Callejas-Cano, Luis M. Delgado, Esther López-Oliva, Conrado Aparicio, Eugenio Velasco-Ortega, Javier Gil

**Affiliations:** 1 Faculty of Dentistry, Universitat Internacional de Catalunya, Josep Trueta s/n, 08195 Sant Cugat del Vallés, Spain; lo962451@uic.es; 2 Bioinspired Oral Biomaterials and Interfaces, Department Ciencia e Ingenieria de Materiales, Escola Enginyeria Barcelona Est, Universitat Politècnica de Catalunya, Av. Eduard Maristany 16, 08019 Barcelona, Spain; darciofonseca@beclinique.pt (D.F.); drcallejas@gmail.com (J.A.C.-C.); conrado.aparicio@upc.edu (C.A.); 3 Bioinspired Oral Biomaterials and Interfaces, Department Expresion Gráfica a la Ingeniería ETSEIAAT, Colom 1, 08222 Terrassa, Spain; luis.m.delgado@upc.edu (L.M.D.); e.lopez@upc.edu (E.L.-O.); 4 ICREA, Institució Catalana de Recerca i Estudis Avançats, Generalitat de Catalunya, 08010 Barcelona, Spain; 5 Department of Stomatology, Faculty of Dentistry, University of Seville, c/Avicena s/n, 41009 Sevilla, Spain; evelasco@us.es

**Keywords:** peri-implantitis, implantoplasty, marginal resection bone, titanium, fatigue

## Abstract

**Background/Objectives**: Peri-implantitis is a common complication affecting approximately 24% of dental implants and is characterized by progressive bone loss and reduced implant stability. Implantoplasty, an intraoral procedure used to remove biofilm by machining the titanium implant surface, has become increasingly common in clinical practice. However, this procedure may compromise the mechanical integrity of implants, especially when combined with peri-implant bone loss, potentially leading to premature fatigue failure. This study evaluated the effect of different marginal bone resection depths, with and without implantoplasty, on the cyclic mechanical behavior of dental implants. **Methods**: A total of 200 commercially pure grade 4 titanium implants were embedded in resin simulating human bone at depths of 3, 4, and 5 mm. A subset of implants underwent implantoplasty with a 0.4 mm surface reduction corresponding to the thread width. Finite element analysis was performed to evaluate von Mises stress distribution and predict fatigue behavior. Numerical results were experimentally validated using a servo-hydraulic MTS Bionix system under ISO 14801:2016 conditions. Fatigue limits were determined from the asymptotic region of the load–cycles-to-failure (S–N) curves, and fracture surfaces were examined by scanning electron microscopy. **Results**: Maximum von Mises stresses were concentrated at the thread–body transition and increased with greater marginal resection depth, with additional stress amplification observed after implantoplasty. Fatigue limits for untreated implants were approximately 351 N, 285 N, and 210 N for 3-, 4-, and 5 mm resections, respectively. Implants subjected to 0.4 mm implantoplasty showed fatigue limits of 311 N, 270 N, and 90 N, respectively. Failure patterns were load-dependent: higher loads produced coronal fractures, whereas lower loads resulted in failure at the implant–abutment connection. Finite element predictions showed strong agreement with the experimental results. **Conclusions**: Excessive marginal resection significantly decreases the fatigue resistance and long-term mechanical reliability of dental implants, particularly when combined with implantoplasty. The main limitations of this study include is in vitro design, the assumptions inherent to the numerical models, and the variability associated with implantoplasty procedures.

## 1. Introduction

**Figure 1:**
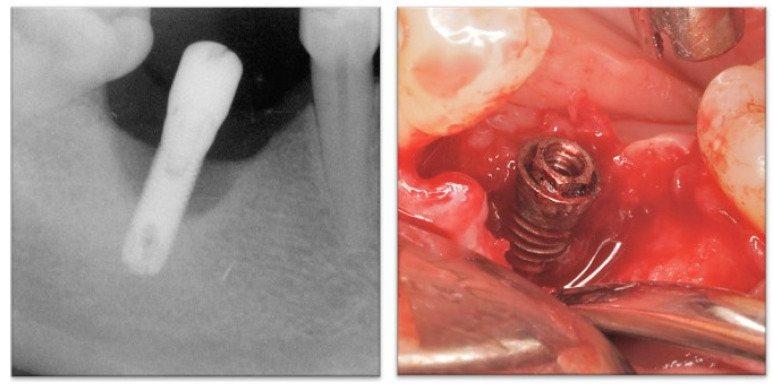
Radiographic and real image of the bone loss effect due to the peri-implantitis disease.

Peri-implantitis is a biofilm-induced inflammatory disease affecting peri-implant tissues, characterized by inflammation of the peri-implant mucosa and progressive loss of supporting bone [1,2,3]. Figure 1 shows the bone loss caused by peri-implantitis. If this bone defect lacks a bony wall around the dental implant, it is virtually impossible to regenerate bone tissue using calcium phosphates. These cases are serious because the implant is held in place by only a small amount of bone and will be subjected to bending forces that can lead to the loss of the dental implant [4].

The management of peri-implant bone loss remains a major clinical challenge, particularly due to the limited predictability of regenerative approaches in contaminated implant surfaces. Several factors, including persistent microbial contamination, implant surface characteristics (e.g., roughness and chemistry), and defect morphology, negatively influence regenerative outcomes [5,6]. In contrast to periodontal tissues, the re-establishment of a functional bone-to-implant interface is difficult to achieve, thereby affecting long-term treatment success [7].

**Figure 2:**
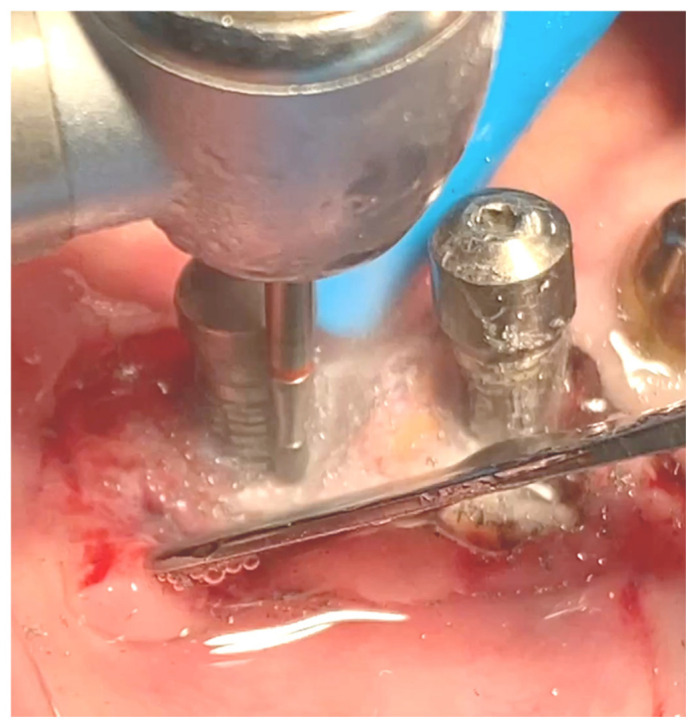
Mechanical cleaning of dental implants to remove biofilms (Implantoplasty).

Effective decontamination of the implant surface is therefore considered a critical step in peri-implantitis therapy. Among the available approaches, implantoplasty has been proposed as a resective surgical technique aimed at mechanically removing the contaminated implant surface through smoothing and polishing procedures (Figure 2). This modification reduces surface roughness and may limit bacterial adhesion, facilitating plaque control [3,8,9]. However, implantoplasty inherently involves the removal of implant material, which may compromise the structural integrity of the implant. In particular, reductions in implant diameter and alterations in surface microstructure have been associated with decreased fatigue resistance and an increased risk of mechanical failure under functional loading [9].

**Figure 3:**
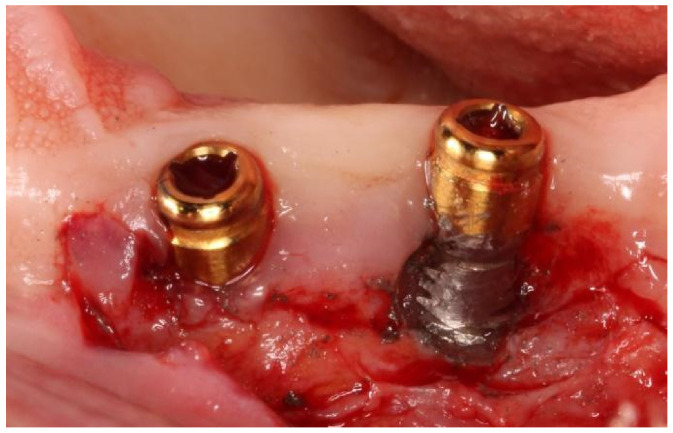
Debris of titanium produced by implantoplasty technique.

One of the effects that most concerns clinicians during implant surgery is the generation of particles ranging in size from nanometers to millimeters (Figure 3). The effect of these particles has been studied by various authors [10,11], confirming their cytotoxicity, especially for dental implants made of the Ti6Al4V alloy. Furthermore, a significant proliferation of inflammatory cells has been observed in the areas surrounding the implant site.

Given these limitations, alternative or adjunctive decontamination strategies have been explored to minimize or avoid the mechanical alteration of the implant surface. Electrochemical cleaning has emerged as a promising approach, enabling the disruption and removal of biofilms through electrolysis-based mechanisms without substantial surface damage [12,13]. Similarly, high-pressure water jet systems and other hydrodynamic techniques have been investigated for their ability to detach biofilms while preserving the original implant topography [14]. Additional methods, including air-powder abrasion, laser-assisted decontamination, and photodynamic therapy, have also been proposed, each with varying degrees of efficacy and impact on implant surfaces [15,16].

Although significant progress has been made, an optimal decontamination protocol has not yet been universally established. Achieving effective biofilm elimination while maintaining the mechanical integrity of the implant continues to represent a major challenge. Consequently, additional research is required to better understand the clinical and biomechanical effects of these therapeutic approaches and to develop evidence-based recommendations for peri-implantitis management.

The novelty of this study lies in the assessment of fatigue behaviour by comparing dental implants with implantoplasty at different levels of bone loss relative to the control group. Clinicians should take these results into account when deciding whether to perform implantoplasty or, if possible, replace the dental implant once the infection has been eliminated. The effect of implantoplasty with different bone resection heights on mechanical behavior under cyclic loads has not been studied, and clinicians need this information to address the various clinical scenarios that may arise. The hypothesis is that implantoplasty and the height of the resection result in a significant reduction in the fatigue life of the dental implant.

The objective of this study is to determine the effect on the fatigue properties of dental implants at different bone resection depths, with and without implantoplasty. This research aims to assist clinicians in making decisions regarding the use of implantoplasty in cases of bone deficiency to prevent implant failure due to fracture.

## 2. Materials and Methods

### 2.1. Samples

**Figure 4:**
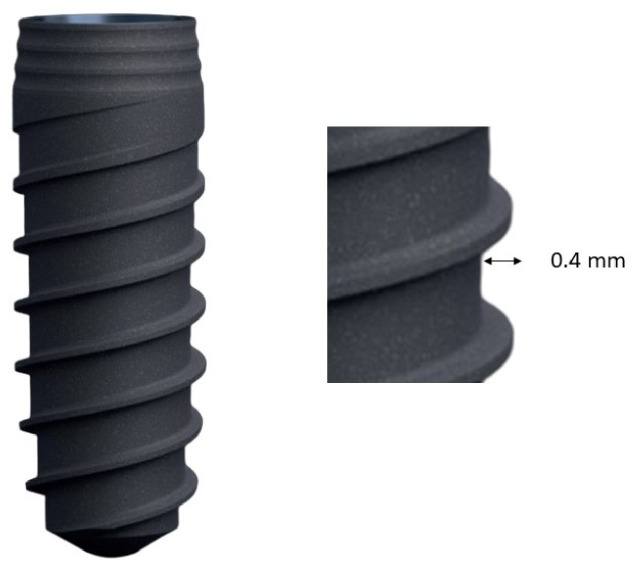
Dental implants studied and scheme of the design of the thread in mm.

A total of 200 dental implants (4 mm diameter; Vega, Klockner, Escaldes Engordany, Andorra) were included in the study. All implants were manufactured from commercially pure grade 4 titanium. The surface roughness (Sa) was 0.9 ± 0.2 μm, and the thread height was 0.4 mm. The implant design and thread geometry are illustrated in Figure 4.

### 2.2. Implantoplasty Procedure

Control specimens were analyzed in their original as-received condition, together with four additional groups subjected to different implantoplasty protocols. To ensure procedural consistency and minimize operator-related variability, all implantoplasty procedures were performed by a single experienced clinician (E.P.G.). A force-controlled dynamometer was used to standardize the applied load during machining. Specifically, a constant force of 10 N was maintained using an automated control system, corresponding to the average force previously recorded from 59 clinicians at the University Dental Clinic of the Universitat Internacional de Catalunya. Each application was sustained for 60 s per sample.

**Figure 5:**
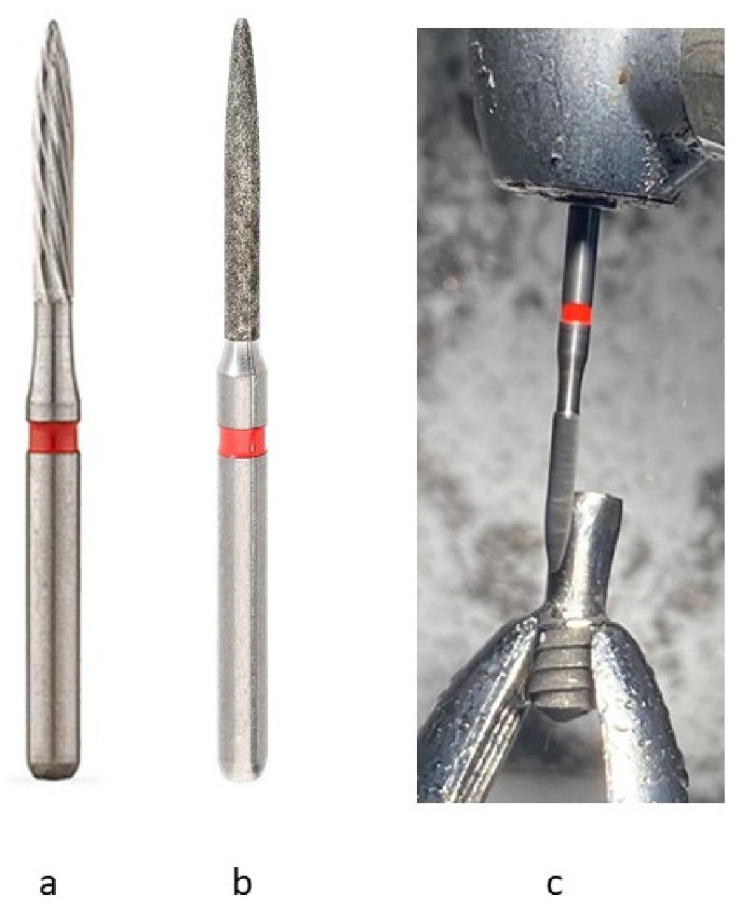
(**a**) Drill of tungsten carbide. (**b**) Drill of fine-particles of diamond to polish the surface. (**c**) Dental implant machining process.

Surface modification was carried out under continuous irrigation using a high-speed turbine (GENTLEsilence LUX 8000B, KaVo Dental GmbH, Biberach, Germany). The procedure consisted of sequential instrumentation with a fine-grit tungsten carbide bur (H379.314.014, KOMET, Lemgo, Germany), followed by polishing with a fine-grain diamond polisher (9618.314.030, KOMET, Lemgo, Germany) (Figure 5). In all cases, the dental implants undergoing implantoplasty were continuously irrigated with demineralized water at 25 °C at a flow rate of 8 mL/s using the Dentsply-Sirona Axano 323 system (Auckland, CA, USA).

Implantoplasty was performed at 0.4 mm depth (corresponding to the original thread height). Dimensional accuracy was verified using a stereoscopic microscope (Zeiss 400Sb-V, Berlin, Germany) with a resolution of 0.01 mm. A total of 120 implants were subjected to implantoplasty and subsequently selected based on achieving the target section reduction. Measurements were performed across the entire surface using a stereomicroscope (Olympus T2400, Tokyo, Japan), and only specimens with deviations within ±10 μm were included in the analysis.

**Figure 6:**
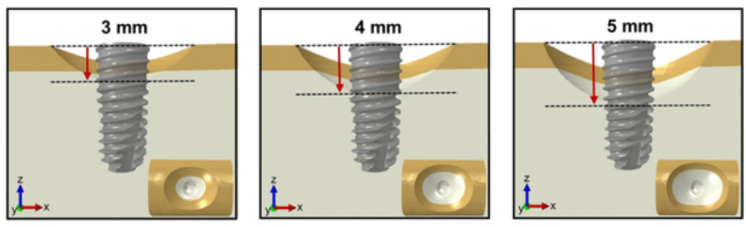
Scheme of the different resection marginal studied.

For the study of marginal bone resorption, three depths of bone loss were examined: 3, 4, and 5 mm. This simulates the various degrees of bone loss that a dental implant might experience. Figure 6 shows this marginal bone resorption.

### 2.3. Finite Elements Simulation

A three-dimensional representation of the 4 mm diameter Klockner Vega implant was provided by the manufacturer. This geometry was later modified using Altair Inspire 2024 in order to replicate both the abutment design used during the in vitro tests and the various implantoplasty conditions analyzed in the study.

Mesh creation and static structural simulations were performed with HyperMesh coupled to the OptiStruct solver (Altair, Troy, MI, USA), whereas fatigue analyses were conducted using HyperLife (Altair, Troy, MI, USA). Before completing the final simulations, a mesh convergence study was carried out to guarantee numerical stability. The finalized finite element model employed 4-node linear tetrahedral elements (CTETRA4) with a global element size of 0.25 mm. In critical regions, especially around the implant threads and zones exposed to maximum bending stresses, local mesh refinement was reduced to 0.05 mm.

To simplify the assessment of bending behavior and stress distribution in the implant walls following implantoplasty, the implant–abutment complex was modeled as a single continuous body with a perfectly bonded interface assumption. Boundary conditions were established by fully fixing all nodes located beneath the embedding level.

Static loading conditions were defined according to the UNE EN-ISO 14801 standard [17]. Following this protocol, an oblique load inclined 30° with respect to the implant axis was applied at a point located 8 mm above the nominal bone level. The embedding plane was positioned 3, 4, and 5 mm apically to this reference level to simulate marginal bone loss due to resection.

For the static analysis, a force of 550 N was applied to calculate the stress distribution later used in the fatigue evaluation. The implant material was considered an isotropic bilinear elastoplastic material characterized by a Young’s modulus of 106 GPa, a Poisson’s ratio of 0.37, and a yield strength of 550 MPa, according to manufacturer specifications. Additionally, a load of 250 N was considered representative of standard masticatory forces. The post-yield material response was represented using a linear hardening model defined through the tangent modulus. This value was obtained from the Ramberg–Osgood relationship assuming a strain hardening exponent of 0.1 and a strength coefficient of 1000 MPa, yielding a tangent modulus of 2765 MPa, which was subsequently incorporated into the finite element model [18,19].

Fatigue life prediction was carried out in HyperLife under the high-cycle fatigue (HCF) approach, since dental implants are exposed to a large number of chewing cycles throughout clinical service. A stress–life (S–N) methodology was adopted. The reference S–N curve for commercially pure grade 4 titanium was generated using an ultimate tensile strength (UTS) of 680 MPa. Because the loading condition followed a pulsating regime (R = 0.1), mean stress effects were incorporated through the Goodman correction model, which considers the detrimental influence of tensile mean stresses on fatigue resistance.

The fatigue damage parameter was defined using the signed von Mises equivalent stress derived from the elastoplastic static analysis, enabling a more accurate representation of the stress state under functional loading conditions.

The Goodman criterion was selected because of its suitability for ductile materials such as commercially pure titanium. In comparison with other criteria, including Gerber or Soderberg, the Goodman approach offers a balanced compromise between conservatism and mechanical reliability. This aspect is particularly important in implant dentistry, where cyclic mastication loads coexist with preload stresses generated during screw tightening. Moreover, incorporating the Ramberg–Osgood formulation into the constitutive material model allowed the simulation to account for strain hardening beyond the elastic range, ensuring that the stress field used in fatigue predictions realistically represented the material behavior under peak loading conditions.

The finite element analysis included several assumptions that should be considered when interpreting the obtained results. One limitation concerns the bone–implant interface, which was assumed to be fully bonded. In reality, osseointegration is incomplete, with bone–implant contact typically ranging between 65% and 70%. Consequently, the simulation may overestimate the actual transfer of mechanical loads. Another limitation is that the model did not consider residual compressive stresses within the implant, which could increase stress levels under mechanical loading. Likewise, potential surface defects such as microcracks or residual drill debris remaining after implantoplasty were not included in the analysis. Such imperfections may alter load transmission and create local stress concentrations that could influence the mechanical response of the implant.

Finally, the finite element results were validated by calculating the regression coefficients of the linear fits obtained from the fatigue test data. MATLAB-Simulink software 2.1 (Natick, MA, USA) was employed for this purpose, specifically using the polyfit and polyval packages, versions 2.0 and 2.2, respectively, to perform the curve fitting procedures and determine the correlation coefficients with the experimental results.

### 2.4. Fatigue Testing

Fatigue tests were conducted under ambient conditions using a servo-hydraulic testing system (MTS Bionix 370, MTS^®^, Eden Prairie, MN, USA) equipped with a 15 kN load cell (MTS 661.19H-03). Hemispherical abutments were connected to each implant using the manufacturer-recommended tightening torque of 35 N·cm. The loading point was positioned 13 mm above the nominal bone level, defined by the resin embedding.

Two-hundred VEGA (Klockner Dental Implants, Escaldes Engordany, Andorra) implants made of grade 4 titanium, which had previously undergone mechanical implantoplasty, were prepared for fatigue testing. These implants were sealed in resin blocks in a precise vertical position, leaving 5 mm of exposed rough surface to simulate horizontal bone resorption of 5 mm (50% of the total implant length), which is 2 mm beyond ISO 14801:2016 specifications.

The threaded portion of each implant is first placed in a small cylindrical metal mold to ensure its vertical orientation and initial stability and then inserted into a larger mold forming a uniform block. Polirresin “Replicas de Superficie” (BCN Qualities S.L., réf. PL-13027, 50 mL, Barcelona, Spain) is poured via an automatic system for precise fixation in 5 min, faithfully reproducing the surface finishes. The complementary epoxy resin MA2+ (PRESI, Cold Resin, 1 kg, Barcelona, Spain) mixed with its PRESI catalyst (100 cm^3^, MA2+ system) is then homogenized and poured using an angular anti-bubble technique around a plasticine bead ensuring the seal. The blocks are left to rest for a minimum of 3 h to ensure complete polymerization/solidification before demolding.

**Figure 7:**
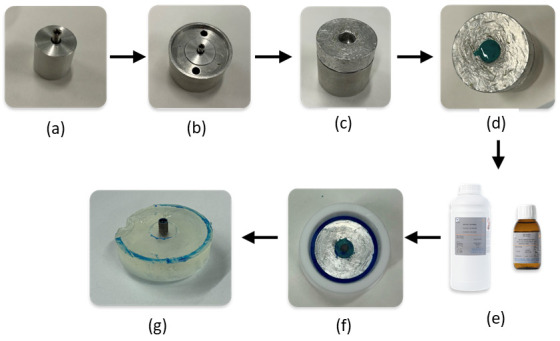
Process of making the study specimens. (**a**) Positioning the implant in the small cylindrical metal mold; (**b**) Inserting this mold into the main metal mold; (**c**) Closing the mold and centering the implant; (**d**) Pouring the “Surface Replicas” epoxy resin; (**e**) Mixing the epoxy resins; (**f**) Applying a layer of modeling clay around the implant and pouring the resin mixture into the mold around the implant; (**g**) Demolding and standardizing the dimensions.

After demolding, the blocks (initial height 12–13 mm) are mechanically planned to 10 mm for perfect homogeneity, guaranteeing rigid fixation without slipping during cyclic loads. This resin (Young’s modulus of elasticity ≥ 3 GPa) simulates alveolar bone, standardizing stress conditions and eliminating anatomical variability for reliable comparative analysis of the mechanical effects of implantoplasty. The preparation process of the fatigue samples can be observed in Figure 7.

This experimental approach is commonly used in dental biomechanics to standardize the test conditions and obtain a homogeneous distribution of stresses around the implant. By molding the implants in the resin, it is thus possible to imitate in a controlled manner the rigidity and mechanical behavior of the alveolar bone during fatigue stresses, while ensuring the reproducibility of the protocols between each sample. This process also facilitates the comparative analysis of the results and ensures that there are no parasitic effects related to the anatomical variability of natural tissues. Thus, the resin filling the mold around the implant makes it possible to faithfully simulate the bone environment and to highlight the phenomena of charge transmission, micro-movement and initiation of damage in clinical situations.

In accordance with the requirements of international standard ISO 14801:2016 [17], each implant was fitted with an abutment and screw specially sized by the manufacturer. This configuration ensures faithful reproduction of the clinical structure of the implant, where the prosthetic abutment acts as a rigid interface between the endosseous implant and the supragingival prosthesis. A torque wrench (Klockner Medical Group, Escaldes Engordany, Andorra) was used, set to the torque recommended by the manufacturer, in accordance with the standard requirements. This control ensures precise tightening, limiting the risk of mechanical play or deformation that could affect the reliability of the tests. The torque wrench is indicated for the surgical and prosthetic phase to tighten the abutments and fixing screws during the final placement of the prosthesis on the implants.

**Figure 8:**
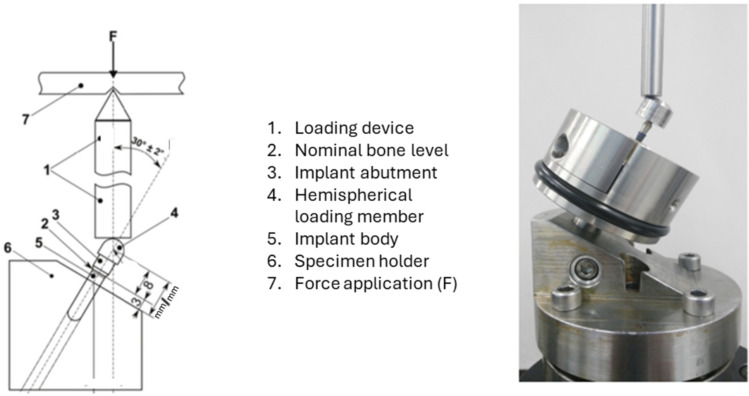
Schematic representation of the test setup according to ISO 14801:2016. In the scheme, point 5 indicates a distance of 3 mm according to the ISO standard, and in this study, to simulate the resection marginal, it is studied for 4 and 5 mm..

Following the ISO 14801:2016 standard [17], specimens were mounted in a stainless-steel fixture with a loading angle of 30° relative to the implant’s longitudinal axis (Figure 8). According to this standard, at least two (preferably three) specimens must be tested at a minimum of four load levels. Additionally, a minimum of three specimens must withstand the number of cycles without failure to establish the infinite life region. Based on these criteria, a minimum sample size of nine specimens was required. In the present study, 20 specimens were tested per experimental group. According to standard practice, the spacing between the original implants must always be at least 3 mm, since these are bone-level dental implants and the standard always requires that this spacing be maintained. This is why the first case is referred to as a control.

It should be noted that, although ISO 14801:2016 [17] simulates worst-case functional loading conditions, it does not aim to predict clinical (in vivo) performance, particularly in cases involving multiple implants supporting a prosthesis.

Each specimen was subjected to a maximum of 5 × 10^6^ loading cycles under uniaxial conditions. The load was applied perpendicular to the tangent of the hemispherical abutment. A load ratio of R = 0.1 was used, with the minimum load set at 10% of the maximum. To reduce machine-induced vibrations, testing was performed at a sinusoidal frequency of 15 Hz. Data acquisition was carried out in real time using TestStar II^®^ software (MTS^®^, Minneapolis, MN, USA).

In accordance with ISO 14801:2016 [17], fatigue testing included at least four load levels. The initial load corresponded to 80% of the maximum compressive force (Fmax), previously determined as 735 N for control implants and 529 N for implantoplasty-treated specimens. Two samples were tested at each load level, using 5 × 10^6^ cycles as the criterion for infinite life. If failure occurred before reaching this threshold, testing was repeated with two new specimens at a reduced load (decreased by 20% when ≥60% Fmax, or by 10% when <60% Fmax).

When two consecutive specimens completed the 5,000,000 cycles without failure, a third specimen was tested under the same conditions. If all three specimens survived, the corresponding load was defined as the fatigue limit. If this limit was identified in fewer than four load levels, additional intermediate levels were introduced by increasing the load in 5% increments.

For each specimen, the number of cycles to failure and failure mode (intact or failed) were recorded. Failure criteria included permanent deformation, loosening of the implant–abutment assembly, or fracture of any component. Results were represented using S–N curves (Wöhler plots), relating the applied maximum load (linear scale) to the number of cycles to failure (logarithmic scale).

### 2.5. Fractographic Analysis by Scanning Electron Microscope

Fractographic analysis was performed using scanning electron microscopy (SEM; JEOL 6400, Tokyo, Japan) combined with X-ray microanalysis (Oxford F300, Oxford, UK).

### 2.6. Number of Samples and Statistical Analysis

Number of dental implants studied: 6 groups × 30 dental implats/group + 20 dental implants rejected due to errors in the implantoplasty procedure, resulting in deviations greater than 10 μm = 200 dental implants.

Of the 30 dental implants for group, 10 were used to determine the flexural strength and the remaining 20 dental implants were subjected to fatigue testing. To determine the sample size, the following method was used: ‘Inference for Means: Comparing Two Independent Samples’ website developed by the Department of Statistics at the Univ. of British Columbia (http://www.stat.ubc.ca/~rollin/stats/ssize/n2.html (accessed 25 February 2025) [20]. A sample size of 7 was calculated for a desired power of 0.80 and significance of *p*-value < 0.05 was. In this work, we tested 10 samples, which is a size larger than the minimum necessary for the power desired. This is because the maximum flexural strength value determines the load percentages at which fatigue tests must be conducted. Given the importance of this value, we increased the number of samples to 10.

Number of samples:

Control—Original without implantoplasty and 3 mm of marginal resection: 30;

Original without implantoplasty and 4 mm of marginal resection: 30;

Original without implantoplasty and 5 mm of marginal resection: 30;

Original with implantoplasty 0.4 mm with 3 mm of marginal resection: 30;

Original with implantoplasty 0.4 mm with 4 mm of marginal resection: 30;

Original with implantoplasty 0.4 mm with 5 mm of marginal resection: 30.

Since fatigue behavior is inherently statistical in nature and several variables may influence the results, including wall reduction caused by implantoplasty, the magnitude of the applied mechanical loads, and procedural variability associated with the operator’s ability to accurately perform implantoplasty, a larger sample size was considered necessary to increase the reliability and confidence of the analysis.

The flexural strength data were statistically analyzed using Minitab version 17 (Minitab Inc., State College, PA, USA). Differences among experimental conditions were assessed using the non-parametric Kruskal–Wallis test and the Mann–Whitney U test. Statistical significance was established at *p* < 0.05.

According to the ISO standard, fatigue testing provides the number of cycles to failure under different cyclic loading conditions, although no specific statistical analysis is required by the standard itself. In the present study, an increased number of fatigue cycles was evaluated to improve the accuracy in identifying the asymptotic region of the fatigue curve and to enable a more precise determination of the fatigue limit. This analysis was performed using the MATLAB-Linear polyval-asymptotic program (Natick, MA, USA).

## 3. Results

The Table 1 shows the results of the elements analyzed in the simulation subjected to maximum stress and their corresponding deformation when force is applied under the conditions described in the methodology.

As anticipated, the highest Von Mises stress values were observed in the implant subjected to a 0.4 mm implantoplasty combined with a 5 mm resection height, reaching 703.4 MPa. This behavior can be attributed to the reduction in implant cross-sectional area together with the increased cantilever effect, both of which substantially weaken the implant structure.

**Figure 9:**
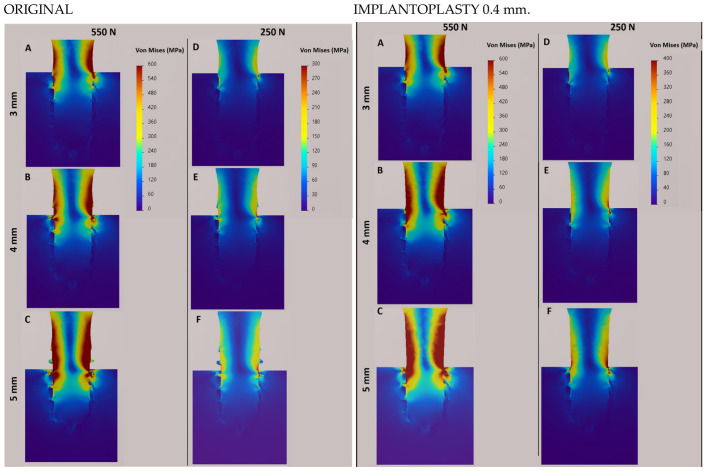
Distribution of Von Mises stress of the two groups: Original and with implantoplasty of 0.4 mm at different resection heights: 3, 4 and 5 mm. Original: (**A**) Load 550 N with 3 mm of resection. (**B**) Load 550 N with 4 mm of resection. (**C**) Load 550 N with 5 mm of resection. (**D**) Load 250 N with 3 mm of resection. (**E**) Load 250 N with 4 mm of resection. (**F**) Load 550 N with 5 mm of resection. Implantoplasty of 0.4 mm: (**A**) Load 550 N with 3 mm of resection. (**B**) Load 550 N with 4 mm of resection. (**C**) Load 550 N with 5 mm of resection. (**D**) Load 250 N with 3 mm of resection. (**E**) Load 250 N with 4 mm of resection. (**F**) Load 550 N with 5 mm of resection.

Notably, the implant treated with a 0.4 mm reduction and a 3 mm resection height showed a maximum stress value (560.7 MPa) very similar to that of the original implant without implantoplasty at the same resection level (566.2 MPa). This finding may be explained by the fact that the 0.4 mm implantoplasty entirely eliminated the upper implant thread, thereby removing the associated stress concentration area. Figure 9 illustrates the Von Mises stress distribution in the different dental implant models subjected to static loads of 550 N and 250 N. In all cases, the highest stress concentrations were located in the transition region between the implant threads and the implant body.

Table 2 shows the flexural strength results for the different batches of implants studied.

**Figure 10:**
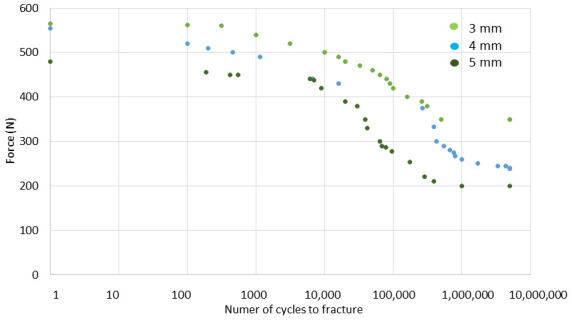
Curve of fatigue for dental implants without implantoplasty at different height resection.

**Figure 11:**
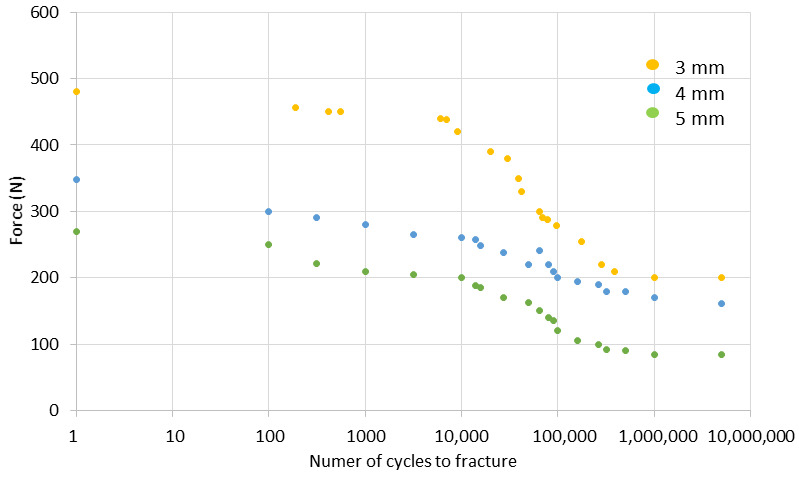
Curve of fatigue for dental implants without implantoplasty at different height resection.

Figure 10 shows the mechanical load curve versus the number of cycles to fracture for dental implants without implantoplasty and with different heights of bone loss. The results indicate that the implants with the lowest bone loss height perform best, followed by those with resection heights of 4 mm and 5 mm. These results were expected due to the cantilever effect exerted during load application. The results are consistent with the maximum flexural strength results in Table 2. Figure 11 shows the fatigue curves of dental implants subjected to 0.4 mm implantoplasty at different bone resection heights. As in the previous analysis, implants with lower bone loss exhibited the longest fatigue life. In contrast, implants with greater bone loss showed the poorest mechanical performance. In these cases, the fracture loads associated with cyclic loading were markedly reduced and may be reached under normal masticatory forces.

The fatigue limits were calculated by plotting the asymptotes based on the values obtained from 5,000,000 cycles in the tests; the fatigue limit is the point where the straight line diverges from the curve. The results can be observed in Table 3.

**Figure 12:**
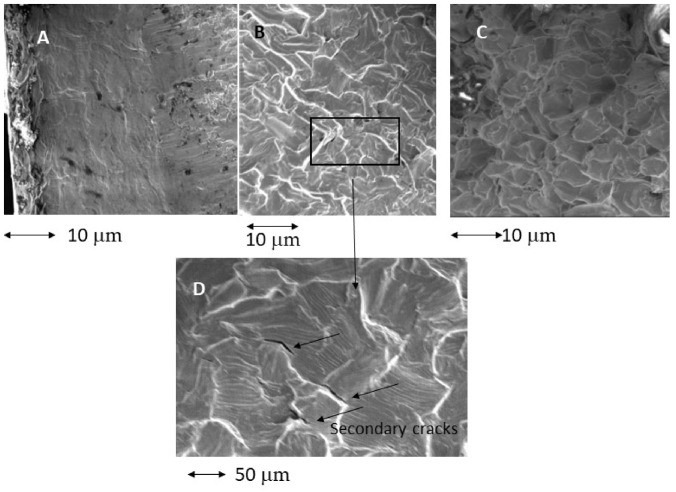
(**A**) Initiation of fatigue crack. (**B**) Propagation of the crack. (**C**) Ductile fracture. (**D**) Propagation zone at higher magnification. It can be observed the crack marks of the advance of the fracture and the presence of secondary cracks.

Fractographic analysis showed that all fractures occurred at the implant–abutment connection. This finding is consistent with the ISO standard, as this region has the smallest load-bearing cross-section. Figure 12 shows representative images of the fracture surfaces. Three characteristic regions can be identified: crack nucleation, crack propagation, and final ductile fracture. The crack nucleation area presents a polished surface caused by friction between the crack faces during cyclic compressive loading. In the propagation region, fatigue striations associated with crack growth are visible. Secondary cracks can also be observed in this area, oriented orthogonally to the main crack propagation direction (Figure 12D). The final region corresponds to ductile fracture failure.

**Figure 13:**
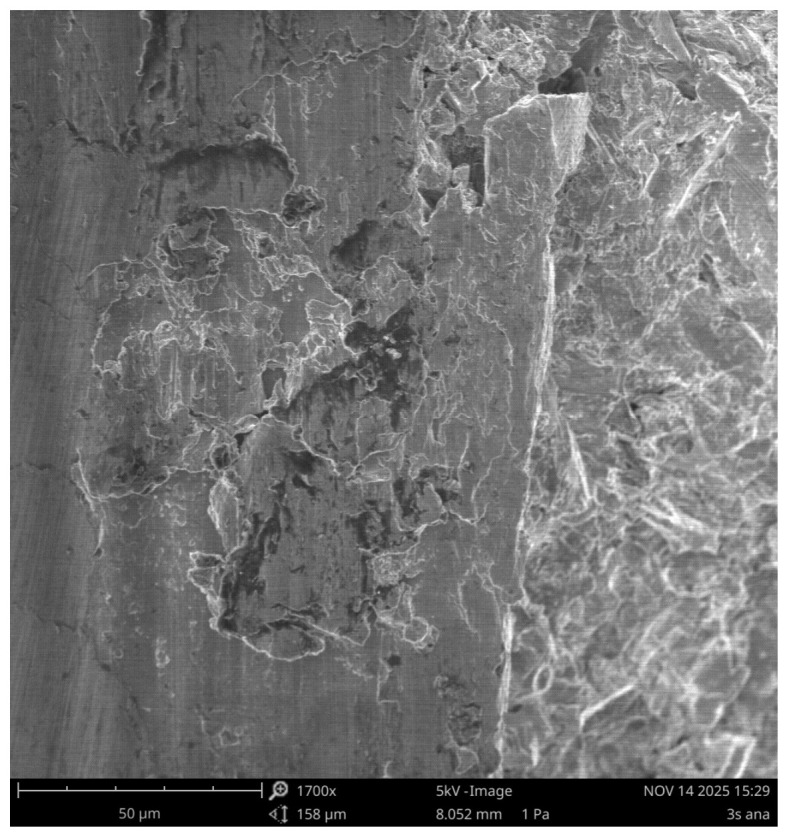
Crack nucleation in the surface of the dental implant in a defect produced by machining.

In fractures observed in dental implants that have undergone implantoplasty, it has been noted that some crack initiations occur in small surface fissures caused by the machining associated with the implantoplasty procedure. These defects cause stress concentration and may promote crack initiation, as shown in Figure 13. This fact represents a limitation in the present study, since the machining is performed manually, which can lead to surface variability.

**Figure 14:**
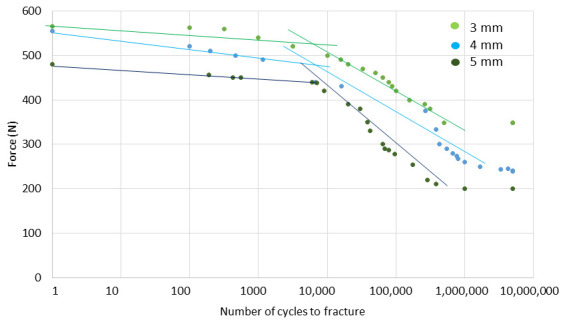
Validation of the finite element simulation of fatigue tests on original dental implants at different resection heights. Linear validation of behavior at different cycle counts using experimentally obtained data.

**Figure 15:**
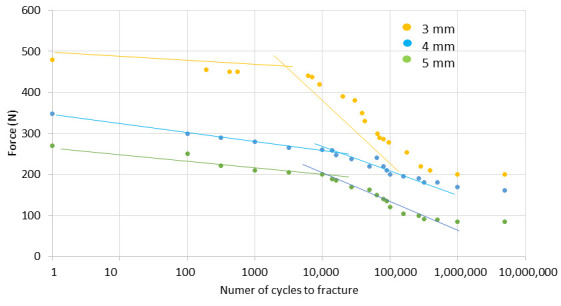
Validation of the finite element simulation of fatigue tests on dental implants with 0.4 mm implantoplasty at different resection heights. Linear validation of behavior at different cycle counts using experimentally obtained data.

Fatigue simulations for dental implants with different resection heights could be approximated by two straight lines, distinguishing between behavior at low cycle counts (up to 10,000 cycles) and a different linear behavior characterized by straight lines with a steeper slope up to the fatigue limit. This can be verified for the control dental implants (Figure 14) and the implants with 0.4 mm implantoplasty (Figure 15) for all resection heights studied.

To determine the validity of this simulation, the linear regression coefficients of the lines obtained from the simulation were calculated in relation to the actual fatigue values obtained in the cyclic tests (Table 4). It was observed that the coefficients are greater than 0.9 in all cases. This indicates that the simulation aligns with the experimental results. However, one must take into account the limitations of finite element simulation, such as the assumption of 100% osseointegration of the dental implant with the bone or the absence of defects on the surface of the dental implant.

## 4. Discussion

As shown by the experimental results, bone loss around dental implants leads to a decrease in maximum flexural strength and fatigue strength. This decrease in mechanical properties is greater with greater bone resection height. This behavior is the same for both original implants and dental implants that have undergone implantoplasty, with the latter showing the greatest loss of mechanical properties compared to the originals. Bone loss around a dental implant result in apical migration of the effective support level, thereby increasing the length of the supracrestal portion of the implant that is exposed to functional loading [21,22,23]. This condition effectively creates a greater cantilever, which significantly alters the biomechanical behavior of the implant system. From a mechanical perspective, the increase in cantilever length leads to a proportional rise in bending moments under occlusal forces. According to basic principles of structural mechanics, bending moment is directly related to the distance between the point of load application and the point of support. As this distance increases, the implant is subjected to higher tensile and compressive stresses, particularly at the crestal bone level and at the implant–abutment interface [24].

This elevated bending moment reduces the structural stiffness of the system and increases its susceptibility to deformation under load. Consequently, the implant exhibits diminished resistance to flexural forces. In addition, the repeated application of these higher stresses during mastication accelerates the initiation and propagation of microcracks within the material, thereby compromising its fatigue strength [25,26,27]. Fatigue failure is particularly relevant in the oral environment, where implants are exposed to cyclic loading over extended periods. The combination of increased stress concentration and reduced cross-sectional support due to bone resorption creates a mechanically unfavorable scenario, ultimately increasing the risk of mechanical complications such as implant fracture, abutment screw loosening, or component failure. In summary, peri-implant bone loss amplifies cantilever effects, leading to increased bending moments, reduced flexural rigidity, and decreased fatigue resistance, all of which negatively impact the long-term mechanical stability of the implant system [28].

One of the surprising experimental findings concerns the fatigue strength values of the original implants and the dental implants that underwent a 0.4 mm implantoplasty, in the case of a 3 mm resection height. It can be observed that the original implants exhibit a longer fatigue life, but the decrease in fatigue strength for those that underwent implantoplasty is not particularly significant. This is because 0.4 mm is the depth of the implant thread, and the connection creates stress concentrations on the surface of the dental implant body that promote the initiation of fatigue cracks. By removing 0.4 mm, this connection disappears, which is why the fatigue life for this modification is the best possible. This phenomenon was studied using finite element analysis by Sevilla et al. and demonstrated for this same type of implant that if the machining depth is 0.2 mm, the fatigue resistance is lower than for a 0.4 mm reduction, since in the first case there would be stress concentration at the junction of the thread and the implant body [29].

Fatigue resistance is lower in implants with implantoplasty due to potential machining defects on the surface that may promote fatigue crack initiation and also reduce the material available for load transfer; however, this is partially offset by the elimination of the stress intensity factor at the thread-implant body connection [30,31,32].

Implantoplasty, in addition to reducing both static and cyclic mechanical properties, presents other drawbacks, including the release of particles and decreased corrosion resistance.

Several investigations have analyzed the same dental implant design with both rough and smooth surface finishes in order to evaluate the influence of surface topography on mechanical behavior [33,34]. These studies demonstrated that implants with sandblasted rough surfaces exhibit a higher fatigue limit compared with smooth-surfaced implants. Specifically, the fatigue limit was reported as 351 N for rough implants and 311 N for smooth implants produced by implantoplasty/machining procedures.

The improved fatigue resistance observed in rough, sandblasted implants, despite sharing the same geometric design as smooth implants, is attributed to the presence of residual compressive stresses generated during the surface treatment process. These compressive stresses hinder the initiation and propagation of fatigue cracks, thereby extending fatigue life. Conversely, smooth implants, whose surface characteristics resemble those of implants treated by implantoplasty, show lower fatigue resistance because they lack the compressive residual stresses induced by abrasive particle blasting [35].

A similar phenomenon occurs after implantoplasty, where titanium machining removes the compressive surface stress layer, consequently reducing fatigue performance. Previous studies by these authors investigated residual compressive stresses in the same implant system using an identical implantoplasty protocol [36,37]. Through grazing-incidence X-ray diffraction employing the Bragg–Bentano method, residual stress values were shown to decrease from −102 MPa in the original implants to −15 MPa following implantoplasty treatment. In addition, the compressive layer generated by alumina sandblasting was estimated to extend approximately 10 µm beneath the surface, indicating that the superficial compressive layer produced by sandblasting was effectively eliminated during implantoplasty. This aspect should therefore be considered when interpreting fatigue life outcomes.

As a result, alternative methods for biofilm decontamination on implant surfaces have been explored. One of these strategies is electrolytic cleaning, where the implant functions as a cathode and hydrogen bubble formation facilitates biofilm detachment from the surface. This electrochemical procedure is performed intraorally and has already been incorporated into clinical practice in certain dental centers. Experimental findings suggest that the technique effectively decontaminates implant surfaces without compromising their mechanical properties. This system, commercially known as GalvoSurge, is progressively gaining acceptance in clinical dentistry [38]. Additional biofilm removal approaches include high-pressure water jet devices and ozone-based systems designed to oxidize and eliminate bacteria associated with biofilm formation [39,40,41].

At present, a debate is emerging: while scientific evidence warns clinicians about the potential adverse effects of implantoplasty, clinical reports suggest favorable outcomes, at least in the short term, although increased inflammatory responses have been observed [42,43,44,45].

From a clinical perspective, the indication for implantoplasty should be carefully evaluated in implants presenting with advanced peri-implant bone loss. The removal of implant material during implantoplasty reduces implant wall thickness and may significantly compromise its mechanical resistance and fatigue behavior. In cases with high bone resection heights, stress concentrations in the exposed portion of the implant may further increase the risk of premature mechanical failure or implant fracture, particularly in narrow-diameter implants or in patients exposed to high occlusal loads [23,46,47,48]. Therefore, the decision to perform implantoplasty should be based on an individualized assessment considering factors such as the extent of bone loss, implant design and diameter, prosthetic configuration, and functional loading conditions. In severe peri-implant defects, alternative therapeutic approaches capable of controlling infection without removing implant material may represent a more favorable strategy to preserve the structural integrity and long-term survival of the implant [49,50,51,52,53].

This study has limitations that should be taken into account. First, the mechanical and fatigue tests were conducted in accordance with the ISO standard, which specifies exactly how to perform them; however, the standard requires that they be conducted at room temperature and without a physiological medium. This fact constitutes a limitation on the results. The implantoplasty protocol commonly used in clinics was also employed, but this always depends on the operator; and although efforts were made in this study to be as objective as possible, there is always a potential for operator-related variation. Another limitation is the use of cement at the implant-bone interface; although it has properties very similar to human bone, there are always differences between living tissue and inorganic material that may affect the final result. Finite element simulation also involves assumptions, such as the assumption that the connection between the bone and the implant is 100%, that is, as if osseointegration were 100%. This data does not align with reality, since normal osseointegration values range between 70% and 80% implant-bone contact.

In any case, the results show consistent behavior, and the fatigue curves fit the model very well for both low and high numbers of mechanical cycles; furthermore, the asymptotes used to determine the fatigue limits have been fitted with linear correlation coefficients greater than 0.9 in all cases. The same applies to the simulated fatigue behavior, yielding straight lines that simulate the fatigue behavior under different conditions and the experimental values. This indicates good validation between the mechanical tests and the in silico simulations.

## 5. Conclusions

Bone resection associated with peri-implantitis significantly compromises the flexural strength and fatigue behaviour of dental implants, and implantoplasty further exacerbates this reduction in mechanical performance. A finite element model of the studied system was successfully developed and validated, enabling the prediction of implant lifespan under different clinical conditions. From a clinical perspective, implantoplasty should be performed with caution in implants presenting with advanced bone loss or high resection heights, as the additional loss of implant material may severely reduce the mechanical properties of the implant and increase the risk of premature fracture. Future research should focus on the development and evaluation of alternative peri-implantitis treatment strategies capable of controlling infection without removing implant material or compromising the mechanical integrity of the implant.

## Figures and Tables

**Table 1:** Flexural resistance and strain associated with the different models studied with the number of elements.

	Elements Analyzed	Von Misses Stress (MPa)	Strain
Original resection 3 mm	518,759	566.2	0.02395
Original resection 4 mm	444,365	571.2	0.02999
Original resection 5 mm	373,916	607.5	0.09555
Implantoplasty 0.4 mm resection 3 mm	353,345	560.7	0.13990
Implantoplasty 0.4 mm resection 4 mm	323,499	678.2	0.08087
Implantoplasty 0.4 mm resection 5 mm	367,890	703.4	0.04576

**Table 2:** Maximum flexural strength for each type of dental implant studied (*n* = 10). Asterisks indicate the statistical difference significance (*p* < 0.05).

	Maximum Flexural Strength (N)
Original resection 3 mm	591 ± 25
Original resection 4 mm	575 ± 13
Original resection 5 mm	482 ± 18 *
Implantoplasty 0.4 mm resection 3 mm	495 ± 22 *
Implantoplasty 0.4 mm resection 4 mm	355 ± 25 **
Implantoplasty 0.4 mm resection 5 mm	280 ± 25 ***

**Table 3:** Fatigue limit results for each group of implants studied, obtained by determining the asymptote of the curve.

	Fatigue Limit (N)
Original resection 3 mm	351
Original resection 4 mm	285
Original resection 5 mm	210
Implantoplasty 0.4 mm resection 3 mm	311
Implantoplasty 0.4 mm resection 4 mm	270
Implantoplasty 0.4 mm resection 5 mm	90

**Table 4:** Linear regression coefficients of the lines obtained by simulation at low and high numbers of cycles for each condition.

	Low Number of Cycles	High Number of Cycles
Original resection 3 mm	0.929	0.988
Original resection 4 mm	0.991	0.920
Original resection 5 mm	0.993	0.919
Implantoplasty 0.4 mm resection 3 mm	0.992	0.983
Implantoplasty 0.4 mm resection 4 mm	0.998	0.976
Implantoplasty 0.4 mm resection 5 mm	0.976	0.967

## Data Availability

The data that support the findings of this study are available from the corresponding author upon reasonable request.
